# Soil nitric and nitrous oxide emissions across a nitrogen fertilization gradient in root crops: A case study of carrot (*Daucus carota*) production in Mediterranean climate

**DOI:** 10.1371/journal.pone.0287436

**Published:** 2023-10-26

**Authors:** Elided Lumor, Udi Zurgil, Ilya Gelfand

**Affiliations:** French Associates Institute for Agriculture and Biotechnology of Drylands, Jacob Blaustein Institutes for Desert Research, Ben-Gurion University of the Negev, Midreshet Ben-Gurion, Israel; Università degli Studi Gabriele d’Annunzio Chieti Pescara: Universita degli Studi Gabriele d’Annunzio Chieti Pescara, ITALY

## Abstract

Insufficient knowledge about soil nitrous and nitric oxide (N_2_O and NO) emissions from vegetable production limits our ability to constrain their atmospheric budget. Carrots (*Daucus carota*) are a globally important, heavily managed and irrigated, high-value horticultural crop. Although intensively fertilized carrots may be an important hot-spot source of N_2_O and NO emissions, we have little information on the response of soil N_2_O emissions to fertilization and no information on the NO emissions response. To fill this knowledge gap, we conducted a replicated field experiment on mineral soil in the Negev Desert. We grew carrots with drip irrigation, applying five fertilization levels, ranging between 0 and 400 kg N ha^−1^. During one growth season we estimated responses of the soil N_2_O and NO emissions, partial crop N balance, and carrot yields to incremental fertilization levels. Carrot yield increased with increasing fertilization from 0 to 100 kg N ha^−1^ and exhibited no further response thereafter. Soil N_2_O and NO emissions were similar at all fertilization levels and did not differ significantly from those in the unfertilized control. The estimated N budget was negative for all fertilization levels. Carrots incorporated 30–140 kg N ha^−1^ into their belowground biomass and 120–285 kg N ha^−1^ into their aboveground biomass per season.

## 1. Introduction

Agricultural soils are an important source of reactive nitrogen (N) gases; nitrous (N_2_O) and nitric (NO) oxides [1, 2]. N_2_O is a potent greenhouse gas (GHG) with global warming potential ~300 times higher than that of carbon dioxide (CO_2_; [2]), and it is the principal cause of ozone-layer depletion [3]. Soil NO emissions are the precursors of tropospheric ozone and can influence local air quality in rural areas [4]. Knowledge of the soil N_2_O and NO fluxes in different eco- and agricultural systems extends our understanding of the overall reactive N gas budget of the atmosphere, allowing the development of emission mitigation measures [5]. However, research into and knowledge of soil N_2_O and NO emissions is not distributed equally worldwide. Drylands receive less scientific attention than areas with more mesic climates, so emissions from drylands are not well constrained [6].

Root vegetables are important food crops, with an overall annual worldwide production of 1002 × 10^6^ Mg, and carrot and turnip (*Daucus carota* and *Brassica rapa*) production totaled 59 × 10^6^ Mg in 2019 [7]. Carrot crops are heavily fertilized, with average recommended fertilization rates between 150 kg N ha^−1^ [8] and >280 kg N ha^−1^ [9, 10]. Globally, root crops use ~2.5 × 10^6^ Mg of N fertilizer annually [11]. Such high fertilization rates can cause high rates of N_2_O and NO emission. However, limited information is available for emissions from root crops in general and from carrots in particular. A recent meta-analysis reported only a handful studies of GHG emissions from root crops, mostly from potatoes [12], and only one study compared multiple fertilization levels [13]. The exudates of root crops secreted into the rhizosphere may enhance heterotrophic microbial and fungal activities and potentially accelerate the soil N and carbon (C) cycles more than grain crops because their carbohydrate concentrations are higher [14]. Although they differ from other vegetable crops in terms of their root structure and carbohydrate metabolism, relatively well-studied potato crops emit, on average, between 0.14 and 1.73% of applied fertilizer as N_2_O (1–3 kg N_2_O-N ha^−1^ year^−1^), depending on the fertilization rate [15], and emissions can increase exponentially with fertilization [16, 17]. Carrots may emit levels that are greater than or equal to those of potatoes, depending upon the specific soil conditions. Unfertilized carrots cultivated on organic soils reportedly emitted 41 kg N_2_O ha^−1^ in the growing season [18]. In contrast, the emissions of fertilized carrots grown in mineral soils (i.e., low in organic matter) were reported to be up to 200 times lower, at 0.2–7.3 kg N_2_O ha^−1^ season^−1^ [19–22]. Given such large variability, it is difficult to set a crop-specific emission factor (EF). This inability to estimate the EFs associated with specific crops or ecosystems limits our ability to constrain the atmospheric gaseous reactive N budget and explain the current trends in atmospheric N_2_O concentrations, which are increasing faster than expected based on known emission sources [23, 24].

Although limited information on N_2_O emissions from carrots and other root crops is available, information on the soil emissions of NO is sparser, even though NO emissions an important pathway of gaseous N loss in drylands [25]. To the best of our knowledge, the only published study of root crops was performed in a potato field in Spain and reported NO emissions of 0.2 kg N ha^−1^ season^−1^, ~30 times lower than the N_2_O emissions from the same soils (7.3 kg N ha^−1^; [22]). Studies of NO emissions from vegetables other than potatoes and carrots (greenhouse cucumbers and field onions) have reported soil NO emissions of 0.6–1.9 kg NO-N ha^−1^ yr^−1^ [26, 27]. No multilevel fertilization study has yet assessed the relationship between increased fertilization and soil NO emissions in root crops. The few existing reports of soil NO emissions from carrots and root crops are insufficient to set EFs for NO for these crops or to propose generalized estimates.

In Israel, most root vegetables, including carrots, are grown in the mineral soils of the northern Negev Desert which represent cropping soils in the south Mediterranean and north Africa region. The carrots are an important commodity; in 2018–2019, Israel produced ~800 × 10^3^ Mg of root crops [7]. Israeli root crops are exported to Europe during the winter growth season and consumed locally in the summer [28]. Carrot farming in Israel is intensive, with recommended fertilization rates of 150–200 kg N ha^−1^ [9, 29, 30]. However, farmers usually apply between 150% and 200% of these recommendations [10].

To determine how incremental fertilization rates affect soil NO and N_2_O emissions, the N balance of the crop, and the carrot yield, we conducted a replicated field experiment on mineral soil in the Negev Desert. We grew carrots with drip fertigation (irrigation + fertilization), applying five fertilization levels ranging from 0 to 400 kg N ha^−1^ in 100 kg N increments. This range included lower than recommended (0–200 kg N ha^-1^) and higher than recommended (300–400 kg N ha^-1^) levels. We measured the soil emissions of NO and N_2_O biweekly, from after sowing but before the start of the fertilization to postharvest and tillage, while fertilizer was added with irrigation every ~5 days. We also estimated the dynamics of inorganic N in the soil, the N uptake by the above- and belowground biomass, and the crop yield and quality. We hypothesized that soil N_2_O and NO emissions increase non-linearly beyond the optimum fertilization levels, whereas the carrot yield is maximum at the optimum fertilization level, beyond which there is no further increase.

## 2. Materials and methods

### 2.1 Study site and soil properties

The study was conducted for one growth season between September 2019 and June 2020 at Kibbutz Urim (31°17’38.0"N 34°32’03.0"E) in southern Israel, a common root crops production area. The soil of the field site was sandy loam, with low C and N contents (Table 1). The area has a semi-arid climate, with highly variable interannual precipitation between September and May. In recent decades (1990–2020), the coldest month has been January, with a mean temperature of 7°C, and the hottest month has been August, with a mean temperature of 33°C. The mean temperature during the experiment was 21 ± 9°C and the total precipitation between September and April was 269 mm, 53 mm above the long-term average of 216 mm (Israeli Meteorological Services, 1990–2020; Besor farm station; [31]).

**Table 1:** Soil properties (0–25 cm layer) at the study site (mean ± standard error [S.E.], n = 12).

pH	Bulk Density*	EC	Clay	Sand	Silt	Organic C	Organic N
	*g cm* ^*-3*^	*Meq L* ^*-1*^	*%*				
8.14	1.60	0.94	13.2	65.5	21.3	2.1 ± 0.1	0.2 ± 0.0

* Bulk density was measured by extracting a core of known volume manually. Bulk density at the 25–50 cm layer was 1.80 kg L^−1^

### 2.2 Experimental design, agricultural management, and baseline sampling

The field experiment was established using a randomized block design, with five fertilization levels including non-fertilized control, lower than standard application of 100 kg N ha^-1^, conservative standard application of 200 kg N ha^-1^ and two high levels of 300 and 400 kg N ha^-1^ (F_0_, F_100_, F_200_, F_300_, and F_400_), each with six replicates (*n* = 6). Apart from the N fertilizer, phosphate (as triple-phosphate) at rate of 400 kg P ha^-1^ and potassium (as KCl) at rate of 300 kg K ha^-1^ were added as per farmers general practice in the area. During the growth season carrots were treated by Racer (flurochloridone; Adama corp), Linurex (linuron; Adama corp), Select (Clethodim; Winflield solutions), and glyphosate (Rodeo; Dow AgroSciences LLC) herbicides; as well as Coragen (Chlorantraniliprole; DuPont) and Vydate (Oxamyl; DuPont) pesticides. The agricultural field used for the experiment was owned by the kibbutz and was managed by rotation of field crops, as is typical in the Negev: carrots (every 4 years), potatoes (every 4 years), peanuts (every 10 years), and wheat during the past decade. After discing (to roughly 25 cm depth) and seedbed preparation, the one-ha field was sprinkle-irrigated with 15 mm (L m^-2^) of water and immediately seeded with carrots (*D*. *carota* var. Nairobi) at a rate of 226 seeds per linear meter of seedbed. The carrots were planted in 4 × 3 rows on raised seedbeds (195 cm wide and ~25 cm high), with two rows of three carrots per the drip line, which is a conventional management practice for carrot farming in Israel (S1 Fig in S1 File). Each replicate had four seedbeds, two for sampling the plants, soil, and gaseous emissions and two for estimating the yield. The N fertilizer was applied by drip fertigation (irrigation + fertilization) as urea–ammonium–nitrate (UAN) solution, 32% N (Haifa Group, Israel). Starting 18 days after sowing (DAS) and during the growth season the field was irrigated every 5–6 days at a rate of 10–20 L m^−2^. Fertilizer addition to irrigation (i.e. fertilization with irrigation or fertigation) began at 24 DAS and ended at 156 DAS. Fertilizer was applied according to the fertilization levels, with equal amounts applied during each fertigation event (i.e., every 5–6 days). The total irrigation during the 2019–2020 growth season was 400 L m^-2^, and together with natural rainfall, the crop received 669 L m^-2^. The fertigation regime was set according to the estimated evapotranspiration rate [32]. All field operations were performed using normal agricultural equipment.

The field samples used to measure the baseline soil N content were collected 23 days before sowing and irrigation, but after preparation of the seedbeds. The soils analyzed for soil properties (Table 1) were sampled from 12 random locations across the one-ha field from the soil layers at 0–25 cm (seedbed) and 25–50 cm depth using a manual auger (5 cm diameter).

### 2.3 Plant sampling and analysis and yield estimation

Plants were sampled from 4 weeks after sowing until they were harvested, from one linear meter of the inner row in the seedbed in each of the six replicates per fertilization level (*n* = 6). Every ~2 weeks, on the day of sampling, the plants were brought to the laboratory, separated into their above- and belowground parts, and weighed. A subsample of four carrots (above- and belowground parts) per replicate (i.e., 24 carrots per fertilization level) was dried in the oven at 60°C until a stable weight was attained, ground to a uniform powder (SM 100 mill, Retsch Gmbh, Germany), and their C and N contents analyzed (CHNS/O analyzer; FlashSmart™ Elemental Analyzer, Thermo Scientific group). To estimate the final yield, one linear meter of two seedbeds (48 rows and 12 linear meters per fertilization level in total) were manually harvested at 183 DAS, a day before the mechanical harvest. To estimate the yield, the carrots were cleaned, sorted by size, and weighed in the field. Part of the yield was designated “unmarketable” in the field. Only the “marketable” yield was used to estimate the carrot size distribution.

### 2.4 Soil sampling and analysis

Every ~14 days, on the day the soil NO and N_2_O emissions were estimated, soil samples were collected from the 0–25 and 25–50 cm soil layers in four experimental blocks (*n* = 4 for each fertilization level). The soil was collected using a manual auger (see above), brought to the laboratory, and sieved (2 mm). Subsamples of the soils were dried at 105°C to estimate their gravimetric water content (g g^−1^). A different set of 5 g subsamples was extracted using 1 M KCl solution (1:5 w:v), filtered (prewashed MN 615 filter, Macherey-Nagel GmbH & Co. Duren, Germany), and analyzed for inorganic N (ammonium, NH_4_^+^ and nitrate, NO_3_^−^) using standard colorimetric methods [33] in a microplate reader (Infinite F50, Tecan, Switzerland).

### 2.5 Measurements of soil NO and N_2_O emissions, flux calculations, and auxiliary measurements

After sowing, starting at 17 DAS (about one week before the start of the fertigation), the soil N_2_O and NO emissions were measured every ~14 days until after harvest and tillage, irrelative to the fertigation. After stop of irrigation, fertilization, and harvest we have measured soil emissions for another ~6 weeks (DAS 190–237) to capture post-season background fluxes. Measurements made more often may be advantageous in rain-feed, one-fertilization-per-season agricultural systems, because the gaseous emissions from soils potentially spike after rain events or fertilization [34–36]. However, the studied system was drip-fertigated, and the soil water content was maintained at the optimum level for plant growth, with no large abrupt changes (S2 Fig in S1 File).

Soil emissions were measured in the field with accumulation (after [37, 38]) and dynamic chambers (after [25, 39]) modified for the site. The description, design, and considerations of the accumulation chamber method used to measure the soil N_2_O fluxes have been given by [40]. The collars used to estimate the soil emissions were firmly fixed into the soil at a depth of ~5 cm, two collars per seedbed (S1 Fig in S1 File) in four blocks (*n* = 4) and were only removed for the mechanical harvest of the carrots. At the time of measurement, the chamber lid was placed on the top of one of the two collars in the seedbed (S1 Fig in S1 File). The collar was made of opaque polyvinylchloride (PVC) tube (19 cm internal diameter [i.d.], 0.5 cm wall thickness, 10 cm height). The aboveground biomass of the plants inside the collars was trimmed to allow measurements to be made. A gas-tight chamber lid was made of the same PVC tubing and was equipped with a vent for pressure equilibration and inlet/outlet valves for the Teflon tubing (0.4 cm i.d., 7 m length) connecting the chamber to the closed path of the quantum-cascade laser analyzer (QCL; GLA151-N2OM1; ABB, Quebec City, Canada). The QCL was used to measure the N_2_O concentration (ppbv or nL L^−1^) by recirculating the headspace air at a rate of ~0.25 L min^−1^. When the lid was placed on the base, the chamber had an average headspace volume of ~3 L and covered a surface area of 0.028 m^2^. The soil fluxes of N_2_O (ng N_2_O-N m^−2^ min^−1^) were calculated from the slope of the linear N_2_O accumulation rate in the chamber headspace, measured every 10 s over period of ~5 min to maximize the linearity of gas production, and corrected for the ambient air temperature (i.e., ~30 data points).

The N_2_O flux (ng N_2_O-N m^−2^ min^−1^) was calculated as follows:

$$F_{N_{2}O}=\frac{a_{v}\times M\times P}{R\times T}\times \frac{V\times 60\frac{s}{min}}{A}$$ (Eq 1)

where a_v_ is the rate of concentration increase (nL L^−1^ s^−1^) in the chamber headspace; M is the molecular weight of N in the N_2_O molecule (28 ng nmol^−1^); P is the atmospheric pressure (1 atm); R is the universal gas constant (0.0821 L atm × [mol K]^−1^); T is the air temperature in°K; V is the volume of gas in the chamber headspace in L; and A is the soil surface area covered by the chamber base (0.028 m^2^).

To estimate the soil NO emissions, a dynamic chamber was placed on the same soil collars as the accumulation chamber (for details see [41]). The lid of the dynamic chamber allowed the free exchange of air between the chamber and the atmosphere while it was connected to the NO–NO_2_–NO_x_ analyzer (42*i* TL; ThermoFisher Scientific). Because the analyzer produces ozone and air cannot be recirculated back to the chamber [39], during the measurements, instead of measuring the change in the NO concentration (nL L^−1^) within the chamber headspace, as is done in the accumulation chamber, the concentration differences between the inlet (i.e., ambient air concentration) and the outlet air from the headspace of the chamber were measured after a steady-state was reached. Measurements were made every 10 s for ~5 min (i.e., ~30 data points). The NO flux (ng NO-N m^−2^ min^−1^) was then calculated as:

$$F_{NO}=\frac{Q}{A}({NO}_{out}-{NO}_{in})\times \frac{M}{R\times T}$$ (Eq 2)

where Q is the flow rate of air through the chamber (2.1 L min^−1^); A is the soil surface area covered by the base (0.028 m^2^); NO_in_ and NO_out_ are the concentrations of NO in the inlet and outlet air (ppbv or nL L^−1^), respectively; M is the molecular weight of N (14 ng nmol^−1^); R is the universal gas constant (0.0821 L atm × [mol K]^−1^); and T is air temperature in°K. Although the NO–NO_2_–NO_x_ analyzer can measure all different NO_x_ species (i.e., NO, NO_2_, and NO_x_), only soil NO emissions are reported because all other species are produced from the oxidation of NO in the atmosphere and are not emitted directly from soils [42] (but see [43] for acidic soils). The emission potentials for NO_2_ and NO_x_ gases were assessed during the data analysis, but no emissions of the gases other than NO that are measured by the analyzer were detected.

The soil temperature (°C) and volumetric water content (L L^−1^) were measured using a GS 3 sensor (ProCheck Decagon Devices, Pullman, WA, USA), according to the manufacturer’s instructions, to a depth of 7 cm at the same time as the gaseous fluxes were measured. We then used the field bulk densities to calculate the water-filled pore space (WFPS) of the upper soil layer as follows:

$$WFPS=(P_{w}\times \frac{D_{b}}{S_{t}})\times 100$$ (Eq 3)

where P_w_ is the water content (ml water g^−1^ soil); D_b_ is the bulk density of the 0–25 cm layer (g cm^−3^); and S_t_ is the total porosity (%) [44].

### 2.6 Data analysis

All statistical analyses were performed using R [45] and SigmaPlot softwares [46]. Analysis of variance (ANOVA) was performed for datasets of normally distributed residuals and homogeneous variance (Shapiro–Wilk and Levene’s tests, respectively), with Tukey’s or Dunnett’s *post hoc* test for treatment comparison. For datasets that violated normality and/or homogeneity of variance (i.e., soil N_2_O and NO fluxes), nonparametric tests were used as follows: the Kruskal–Wallis test for independent samples, with the Bonferroni multiple comparison test; and Friedman’s (F) test for repeated-measures data, with the Wilcoxon matched pairs test for comparisons. Figure error bars represent standard errors (S.E.). The soil N_2_O emissions were less variable than the NO emissions, whereas NO showed more extreme outliers. No data on the soil gaseous emissions was omitted from the analysis. Differences were deemed statistically significant at *p* < 0.05.

The partial seasonal N balance of the carrot crop was estimated from the sum of the N flows and pools: fertilizer N input; initial soil N pool (measured in the field before the start of fertilization and corrected for sampling depth and soil bulk density); N uptake by carrots (estimated based on the dry weight yield); aboveground to belowground biomass ratio and N content of carrot plants (estimated from subsamples taken on the day of harvest [section 2.3]); and the cumulative soil N emissions until the day of tillage after harvest (237 DAS), as follows:

$$N\;balance\;(kg\;N\;ha^{-1}\;season^{-1})=F_{N}+INI_{N}-AG_{N}-BG_{N}-N_{2}O_{N}-NO_{N}$$ (Eq 4)

where F_N_ is the N supplied with fertilization; INI_N_ is the initial soil N pool (0–25 cm layer); AG_N_ and BG_N_ are the N removed by above- and belowground biomass, respectively, calculated from the manually harvested root yield data, the below- to aboveground biomass ratio, and the plant N content estimated from subsamples; and N_2_O_N_ and NO_N_ are the cumulative soil gaseous N emissions between sowing and harvest (all in kg N ha^–1^). The soil emissions of ammonia (NH_3_) and gaseous N_2_, as well the NO_3_^-^ leaching were not assessed in the current study, but they may contribute to the total N budget. Therefore, we underestimated the N loss from the system and our partial N balance is less negative than it could be if all losses were included.

The cumulative soil gaseous N emissions of N_2_O-N and NO-N were calculated using linear interpolation between sampling dates [47–50]. To estimate the seasonal cumulative emissions, the fluxes measured biweekly using individual chambers between 17 DAS and 237 DAS were used (S3 Fig in S1 File).

Nitrogen use efficiency (NUE) was estimated as agronomic efficiency (A_E_) as follows:

$$A_{E}(kg\;yield\times kg^{-1}\;N\;applied)=\frac{Y_{X}-Y_{0}}{N_{F}}$$ (Eq 5)

where *N*_*F*_ is the N fertilizer applied (kg N ha^−1^); *Y*_*X*_ is the yield under a specific fertilization treatment; and *Y*_*0*_ is the yield with no fertilization (kg fresh yield ha^−1^).

## 3. Results

### 3.1 Carrot yield, quality, and N concentrations

**Fig 1:**
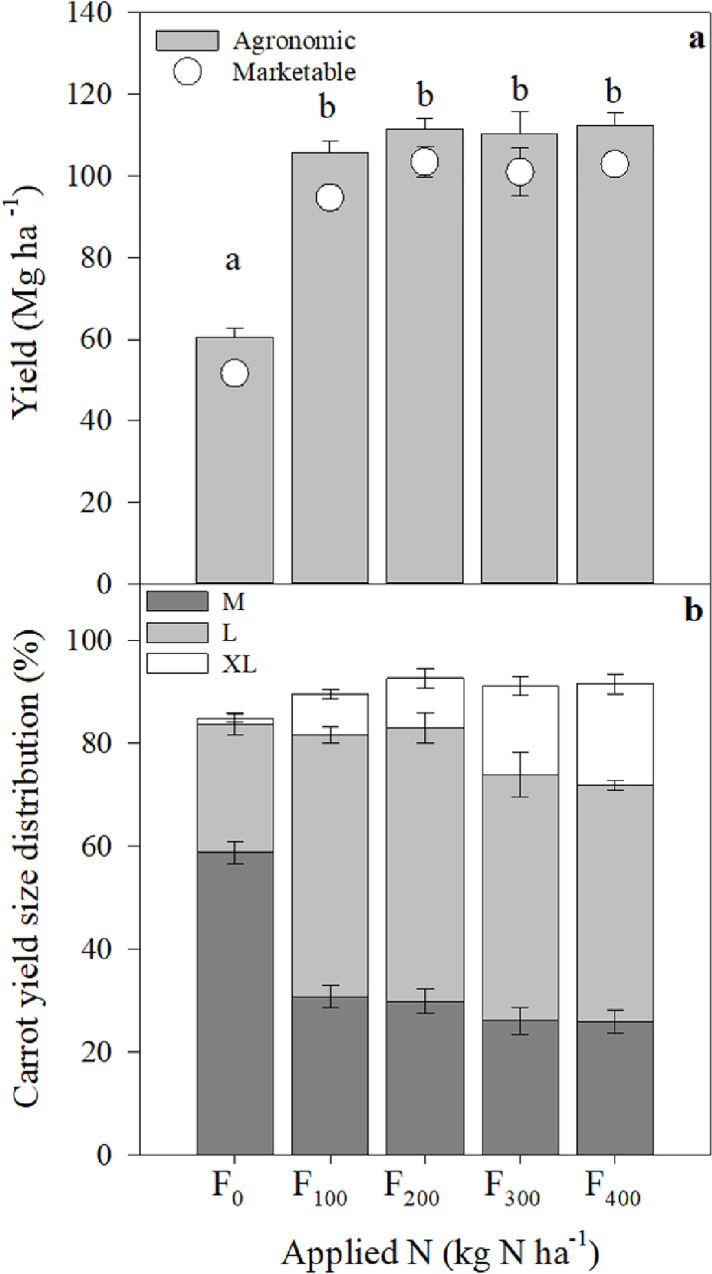
Fresh (**a**; bars) and marketable (**a**; open symbols) yields, all in Mg fresh yield ha^−1^, and fresh yield size distribution (**b**; %): medium (M), large (L), and extra-large (XL) carrots grown under different fertilization regimes; means ± S.E. (*n* = 5). Statistically significant differences are indicated by lower-case letters (ANOVA; F_4,25_ = 45.6, *p* < 0.05). For statistical analysis of the size distribution (panel **b**), refer to S2 Table in S1 File. The size distribution does not sum to 100% because part of the yield was unmarketable.

The fresh and marketable yields of carrot (taproot) increased significantly between F_0_ and F_100_ but did not change with higher levels of fertilization (Fig 1A). However, the size of the carrots in the marketable yield category increased continuously with increasing fertilization (Fig 1B). Without fertilization, 59% ± 5% of carrots were of medium (M) size, 25% ± 5% were large (L), and 1% ± 2% were extra-large (XL). With increasing fertilization levels, the percentages of L and XL carrots increased. At intermediate fertilization (F_100_ and F_200_), ~52% of the yield was L and ~9% XL. At high fertilization levels (F_300_ and F_400_), the percentage of XL carrots increased to ~19% and was significantly larger than the percentage of XL carrots at all other fertilization levels (Fig 1B; S1 and S3 Tables in S1 File). At all but the F_0_ fertilization level, ~7% of the total yield was unmarketable; at the F_0_ fertilization level, the unmarketable yield was twice that, at 14% (Fig 1B; S1 and S2 Tables in S1 File).

**Fig 2:**
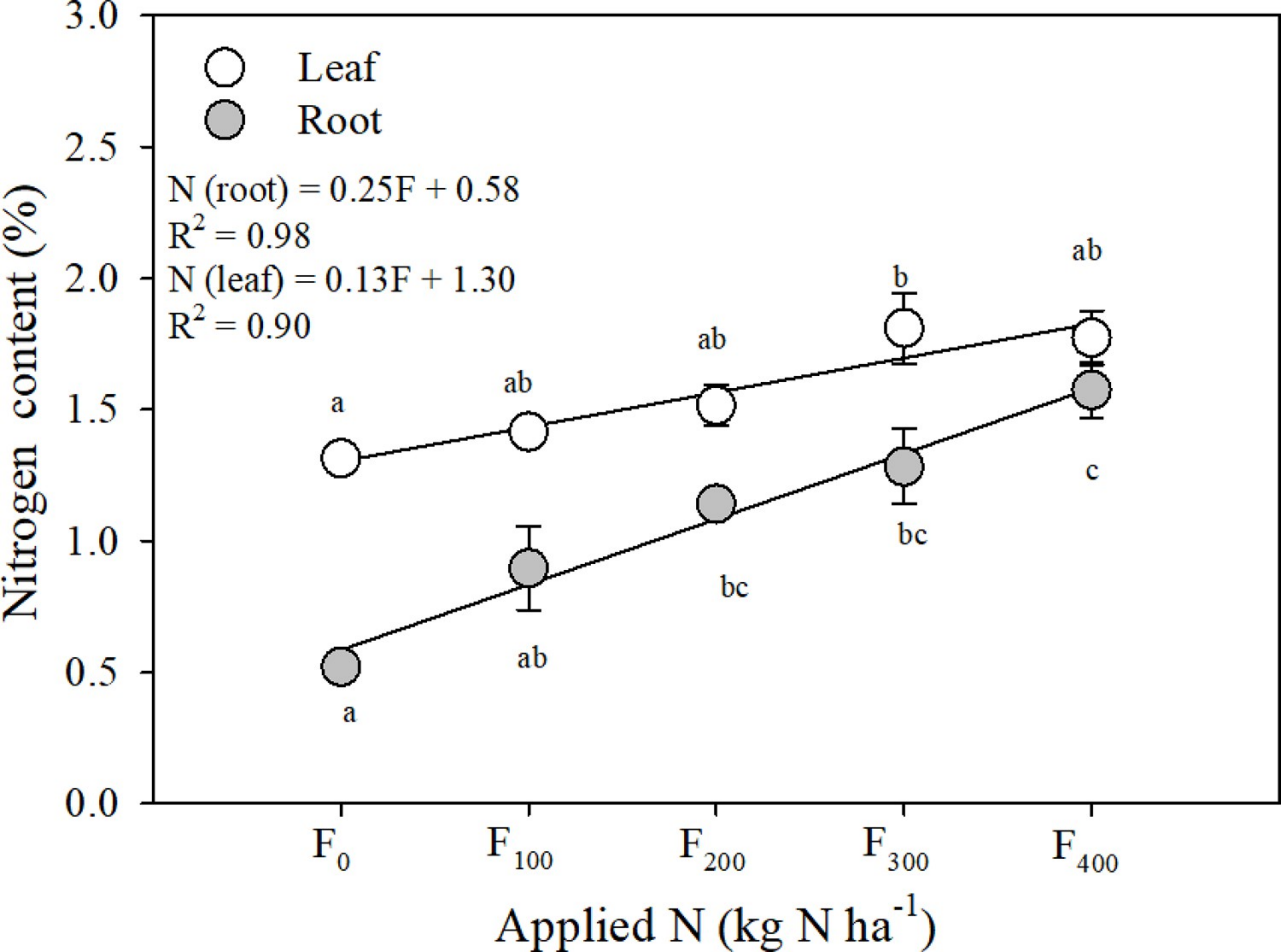
Effect of increasing fertilization levels on above- and belowground biomass N concentration (%) in carrots; mean ± S.E. (*n* = 5). Statistically significant differences are indicated by lower-case letters. For leaf: ANOVA F_4,15_ = 3.899, *p* = 0.023; for root: ANOVA F_4,15_ = 8.780, *p* < 0.05.

The nitrogen contents of both the aboveground and belowground biomass (i.e., leaves and taproots, respectively) of the harvested carrots increased linearly with the fertilization level (Fig 2). Carrots grown without fertilizer had significantly lower N content than fertilized carrots, and the highest N content was measured in carrots grown with the highest fertilizer input. In the taproots, the N content increased at a rate of 0.25% × 100 kg^−1^ N applied, from 0.5% ± 0.0% in the unfertilized carrots to 1.6% ± 0.1% in the carrots fertilized with 400 kg N ha^−1^. In the leaves, the increase was half that in the roots and ranged from 1.3% ± 0.1% at F_0_ to 1.8% ± 0.1% at F_400_ (Fig 2).

### 3.2 Dynamics of soil inorganic N

**Fig 3:**
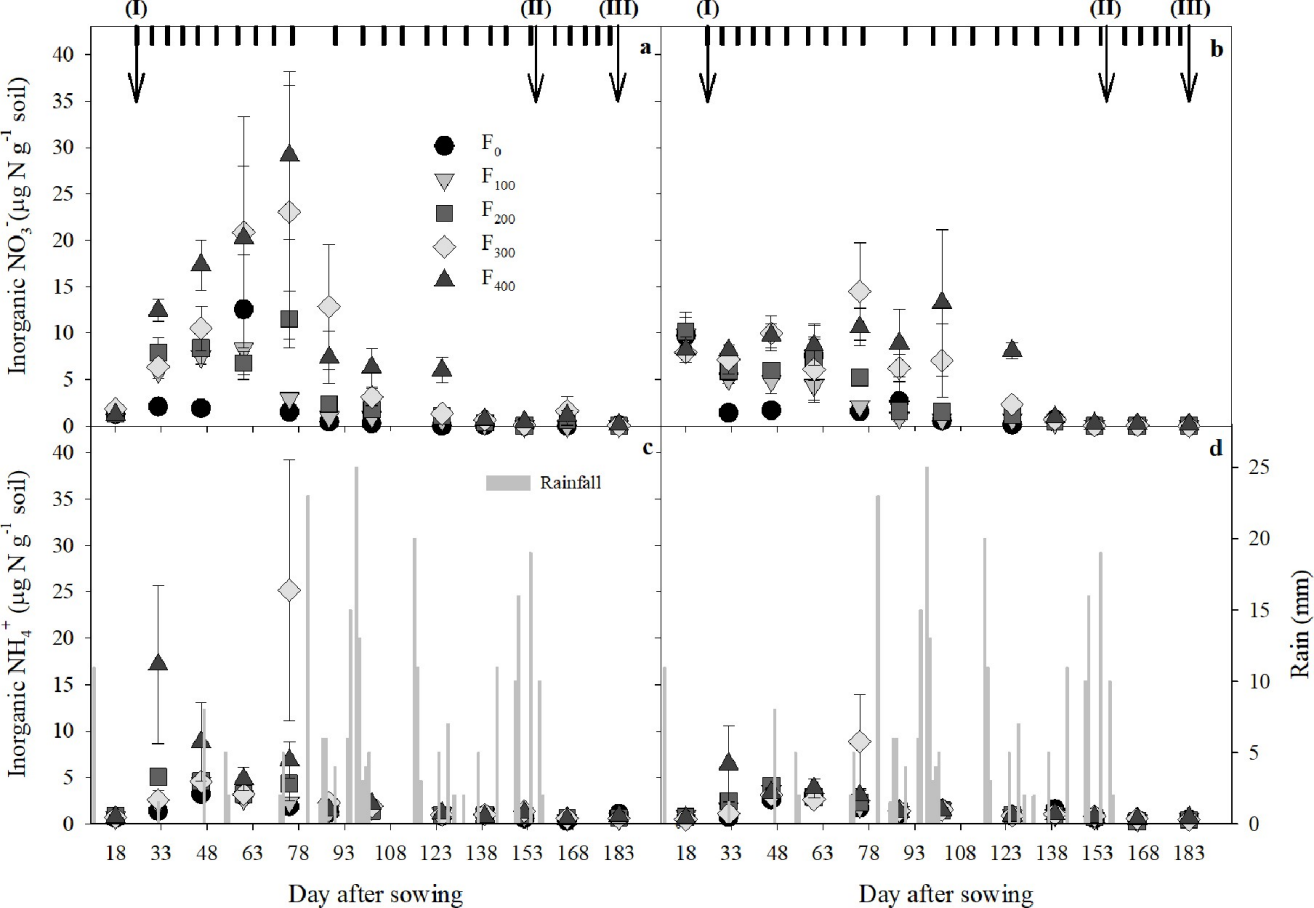
Dynamics of soil inorganic N pool (μg N g^−1^ soil) during carrot growth season; mean ± S.E. (*n* = 4). Nitrate: **a** (0–25 cm; seedbed), **b** (25–50 cm; below seedbed); and ammonia: **c** (0–25 cm; seedbed), **d** (25–50 cm; below seedbed). After harvest at 183 DAS, the seedbeds were overturned and tilled and the N contents in both layers (seedbed and below the seedbed) were homogenized. Soil N content after tillage was measured at 237 DAS. Inorganic N concentrations were 2.11 ± 0.56 μg N g^−1^ soil (NO_3_^−^) and 0.58 ± 0.12 μg N g^−1^ soil (NH_4_^+^) and did not differ significantly between different fertilization levels. Start of fertilization (i), end of fertilization (ii), and harvest (iii) are shown with arrows. Irrigation events are shown by marks in the upper panels. Rainfall is presented in the lower panels (identical in both lower panels).

At the beginning of the season, before sowing, the inorganic N contents of the soils were uniformly low, with slightly higher NO_3_^−^ concentrations at the surface (0–25 cm soil layer). With the commencement of fertigation, the inorganic soil N content in the seedbed soil gradually increased, with NO_3_^−^ peaking at up to ~30 μg N g^−1^ soil at about 2 months after sowing (Fig 3A). Beneath the seedbeds (25–50 cm soil layer), the highest measured NO_3_^−^ concentration was ~10 μg N g^−1^ soil, with no pronounced peak (Fig 3B). Both layers of soil had low inorganic N contents by 138 DAS and thereafter, despite the continuous supply of N by fertigation (Fig 3A and 3B). The ammonium concentrations in the soil were overall lower than the NO_3_^−^ concentrations, at ~ 5 μg N g^−1^. The soil NH_4_^+^ content peaked about one month earlier than the NO_3_^−^ content and was depleted around 30 days earlier (Fig 3C and 3D), despite the continues use of the UAN solution for the fertigation.

### 3.3 Soil N_2_O and NO emissions

**Fig 4:**
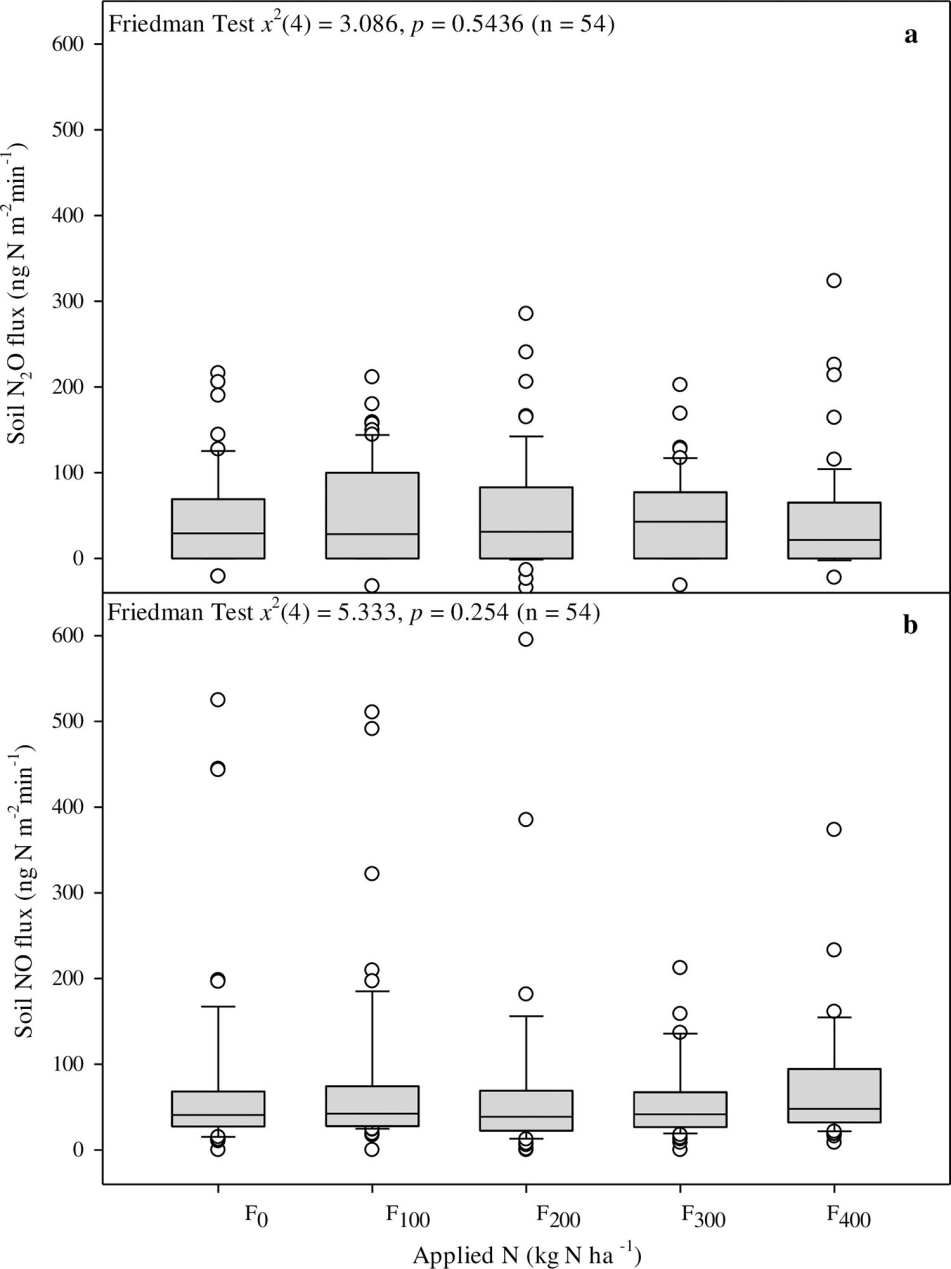
Effects of increasing N fertilization levels on soil emissions of N_2_O **(a)** and NO **(b)** (individual measurements, ng N m^−2^ min^−1^; *n* = 4). No significant differences were detected.

**Fig 5:**
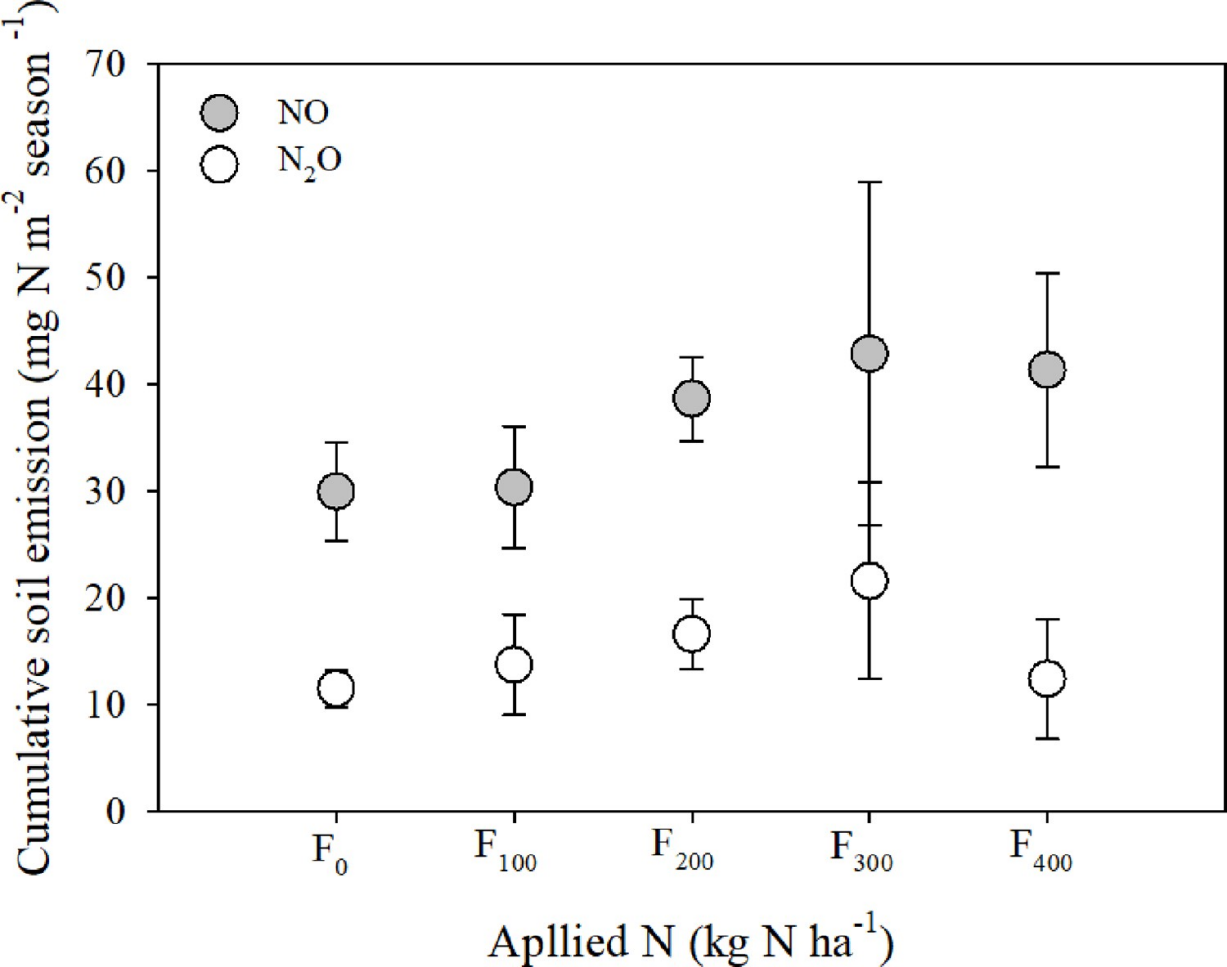
Effects of increasing N fertilization levels on cumulative soil emissions of N oxides (mg N m^−2^ season^−1^). No significant differences were detected.

No significant differences in soil N_2_O and NO emissions were detected across all fertilization levels (Fig 4, S3 Fig in S1 File). Both gases showed highly skewed emissions, with average fluxes below 100 ng N m^−2^ min^−1^ and numerous outliers of several 100 ng N m^−2^ min^-1^ (Fig 4A and 4B). The skewness of the measured N_2_O emissions ranged from 0.4 to 1.3, whereas the skewness of the NO emissions was ~3 times larger, between 3.2 and 6.0 (S3 Table in S1 File). However, the coefficient of variation was similar between the two gases, ranging from 1.1 to 2.7 (S3 Table in S1 File). Of the 300 individual fluxes measured over the growth season, 8% of soil N_2_O emissions were negative and 30% were zero (i.e., no detectable flux). For a detailed explanation of the decision tree for the N_2_O emissions analysis, see [40]. In contrast, the NO emissions were 99% positive (i.e., emission to the atmosphere) and only 1% of emissions were zero (S4 Table in S1 File). No correlation between soil WFPS and the soil N_2_O or NO fluxes were detected (S4 and S5 Figs in S1 File). However, both gases had a pronounced optimum soil temperature, with highest emissions at 22–28°C and decreasing thereafter (S4 and S5 Figs in S1 File). The cumulative seasonal emissions calculated using linear interpolation between the measurement fluxes of each individual replicate were higher for NO (30–43 mg N m^−2^ season^−1^) than for N_2_O, (12–22 mg N m^-2^ season^-1^; Fig 5), but they did not differ significantly across fertilization levels, including the unfertilized treatment. The cumulative emissions of soil NO and N_2_O were 0.3–0.4 and 0.1–0.2 kg N ha^−1^ season^−1^, respectively (Table 2). These low emissions resulted in low emission intensities for the carrot crop, of 1.10–1.96 g N_2_O–N Mg^−1^ fresh yield (Table 3).

**Table 2:** Partial seasonal N balance (kg N ha^−1^) of the carrot crop (mean ± standard error [S.E.], *n* = 4).

Fertilization level	N input	Initial soil N pool*	Aboveground biomass uptake†	Belowground biomass uptake†	Soil N_2_O emission**	Soil NO emission**	Final soil N pool††	Balance#
	*kg N ha* ^*-1*^							
F_0_	0	55.0 ± 6.9	119.2 ± 5.1	57.1 ± 4.9	0.12 ± 0.02	0.30 ± 0.05	4.7 ±1.6	-121.2 ± 7.1
F_100_	100	55.0 ± 6.9	175.1 ± 7.0	121.4 ± 21.6	0.14 ± 0.05	0.30 ± 0.06	2.0 ± 0.4	-141.5 ± 22.7
F_200_	200	55.0 ± 6.9	204.4 ± 10.2	159.7 ± 4.9	0.17 ± 0.03	0.39 ± 0.04	2.7 ± 0.8	-109.2 ± 11.3
F_300_	300	55.0 ± 6.9	284.5 ± 21.2	175.7 ± 19.6	0.22 ± 0.09	0.43 ± 0.16	2.6 ± 0.3	-105.4 ± 28.9
F_400_	400	55.0 ± 6.9	276.7 ± 16.0	208.7 ± 14.0	0.12 ± 0.06	0.41 ± 0.09	4.1 ± 0.6	-30.4 ± 21.3

* Soil inorganic N pool measured after initial seedbed formation on 22 September 2019, ~3 weeks before sowing

** Cumulative soil N oxide emissions used to calculate the N balance estimates until the day of harvest

† Based on the dry mass; carrot water content was 86.5% ± 1.1% and was not affected by fertilization level

†† Soil inorganic N pool measured at harvest before tillage of seedbeds, not included in the net N balance

# See Section 2.6

**Table 3:** Nitrous oxide emission intensities and agronomic efficiency of carrot production (g N Mg^−1^ fresh yield^−1^).

Fertilization level	Emission intensity	Agronomic efficiency (Ae)*
	*g N*_*2*_*O-N Mg*^*-1*^ *yield* ^*-1*^	*kg yield kg*^*-1*^ *N*
F_0_	-	-
F_100_	1.30	451
F_200_	1.49	255
F_300_	1.96	166
F_400_	1.10	130

* Calculated based on the agricultural fresh yield for marketing (Section 2.3)

### 3.4 Nitrogen balance, agronomic efficiency, and N_2_O emission intensity of the carrot crop

The carrots cultivated on mineral soils had a negative N balance of between -30 and -141 kg N ha^−1^ season^−1^, irrespective of the fertilizer applied. The unfertilized control acquired ~176 kg N ha^−1^ from soils, with an overall budget of -121 kg N ha^−1^, which did not differ from those of the fertilized treatments, except at the highest rates of N application (F_300_ and F_400_). However, the N balance at the highest fertilization level (400 kg N ha^−1^) was close to zero, with a total budget of -30 ± 21 kg N ha^−1^ season^−1^. The total N uptake to the above- and belowground biomass under the F_400_ treatment was 485 kg N ha^−1^ (Table 2). The N content of the aboveground biomass, estimated to be 119–284 kg N ha^−1^ across all fertilization treatments, was left in the field after harvest, contributing to future mineralization and the renewal of the soil N pool (Table 2). The belowground biomass of the fertilized carrots removed 121–209 kg N ha^−1^ during harvest, whereas the unfertilized crop removed 57 kg N ha^−1^ because the yield was almost two times lower (Fig 1 and Table 2). Both the NO and N_2_O emissions made little contribution to the overall N balance of the crop. The high N uptake efficiency of the carrots resulted in high agronomic efficiency (Ae), of 130–451 kg yield kg^−1^ fertilizer N, with the highest Ae at F_100_ and the lowest at F_400_ (Table 3).

## 4. Discussion

The results of our field experiment, in which N fertilizer was added incrementally to carrot crops by drip-irrigation (fertigation), did not show an increase in soil NO and N_2_O emissions with increasing fertilization past the optimal threshold. Surprisingly, the emissions also did not differ significantly between the unfertilized and fertilized treatments. Moreover, the carrot yield was not affected by the increase in the N fertilization rate, and the only difference was observed between the fertilized and unfertilized soils. However, fertilization strongly influenced the size distribution of the yield and the N content of the above- and belowground biomass, which increased linearly with the N application rate.

### 4.1 Carrot yield and N content changes with fertilization

The lack of a yield response to the application of fertilizer above 100 kg N ha^−1^ (Fig 1) in carrot crops was reported earlier and has been observed in carrots grown on both mineral and organic soils [8, 9, 51–53]. However, an increase in the N content of the carrot biomass indicated an increase in the N uptake of the crop with fertilization, which did not result in an increase in yield (Fig 2). The optimum fertilization level for maximum yield found here, 100–200 kg N ha^−1^, is similar to those reported earlier [54–56]. The highest agricultural N use efficiency observed with fertilization at 100 kg N ha^−1^ (Table 3) also indicated that an application of <200 kg N ha^−1^ is optimal. However, the yield measured here was at the high end of earlier reports of 70–100 Mg fresh yield ha^−1^ [53, 56–58]. The high yield measured here can be attributed to the climate and the management of the experimental field. Drip fertigation, which directly delivers fertilizer to the vicinity of the plant, together with warm temperatures during the growing season and loamy soils, may have created optimum conditions for carrot growth [59]. A relatively high percentage of the yield (87%–93%) was of marketable quality (Fig 1), higher than the ~80% reported previously [53]. The strong effect of fertilization on the size distribution of the carrots (e.g., M vs L vs XL; Fig 1B) and increasing with amount of applied fertilizer N content of carrots pointing on other, than biomass increase responses, such as increasing inorganic N content of the crop (i.e., NO_3_^-^ accumulation in carrots). Such high N uptake not always beneficial, for example, high N uptake would reduce the quality of the crop because the shelf-life of overfertilized carrots is limited [60].

### 4.2 Seasonal dynamics of soil inorganic N

The inorganic soil N pool increased after the start of fertigation and before the onset of intensive N uptake by the crop (Fig 3A and 3B), which was estimated to start at ~70 DAS [61, 62]. Consistent with earlier reports, we found very low inorganic N concentrations after ~100 DAS in the seedbeds and 25–50 cm soil layers (Fig 3), indicating the efficient uptake of N by the carrot root systems. This was attributable, in part, to the delivery of low concentration of soluble N to the vicinity of plants by the fertigation process, in contrast to other fertilization methods, as pre-sowing fertilization. The overall low N concentrations in the soils and the co-occurrence of peak inorganic N concentrations in the 0–25 and 25–50 cm soil layers (Fig 3) indicate a low down-movement of inorganic N in the soils and a small loss of N beneath the root zone by leaching. A low potential N loss is also supported by the high rate of N removal by the crop (Table 2).

### 4.3 Effect of increasing fertilization on soil N_2_O and NO emissions

Surprisingly, we found no effect of fertilization on the soil N_2_O and NO emissions, contrary to earlier reports of root vegetables, vegetables, and other crops [16, 26, 63]. Moreover, the emissions of both N_2_O and NO were low (Fig 5), despite the optimal WFPS for both gases (40%–75%) during most of the growing season (S2 Fig in S1 File; [64]). The low emissions of N gases found in our study can be attributed to fertilizer application. Fertilizer application by drip irrigation is delivering small portions of N in the vicinity of the crop reducing potential of loss [59]. Although low, the N emissions were highly skewed, and the coefficient of variation was high (S3 Table in S1 File), as expected for soil emissions measured in agricultural fields [48]. The NO emissions were 3–4 times more skewed than the N_2_O emissions and both gases showed high variability (S3 Table in S1 File). The NO emissions reported here were at the lower end of emissions previously measured in high-temperature agroecosystems [4]. Contrary to expectation [64, 65], neither the NO nor N_2_O emissions correlated with WFPS (S4 and S5 Figs in S1 File), but they correlated with soil temperature, as reported by [64], with the highest fluxes measured at 22–28°C. The laboratory incubation of similar soils from the Negev Desert showed a comparable emission optimum for NO [66], but there has been little research on the NO emissions in carrot crops and other agroecosystems in Israel or elsewhere.

The comparison of the N_2_O and NO emissions with and without fertilization generated trivial seasonal EFs of 0.00%–0.13% of applied fertilizer for both gases. Such low fluxes can be attributed to the fertigation of mineral soils supporting crops with low C contents (Table 1) and high N uptake efficiency, which effectively remove the inorganic N pool from the soil, as reflected in the negative N balances (Table 2). The low emissions of NO and N_2_O measured here reported previously for fertigated dryland agroecosystems [59, 67] and indicate the potential for the mitigation of soil N_2_O emissions in dryland agriculture by drip fertigation. The high degree of control of soil moisture and the time of fertilizer application permitted with fertigation, as well as the delivery of N close to the plant roots, seems to maximize N utilization.

### 4.4 Negative N balance

While there are limitations to the N balance presented here, and we have not included such potentially important pathways of N loss as soil N_2_ emissions and NO_3_^-^ leaching. And there is a need to include these for a more comprehensive N budget for the root crops in the future. The partial N balance presented here can still provide a useful initial assessment of N relations in the ecosystem under study. The negative partial N balances calculated here indicate the efficient N uptake by carrots. We have not measured soil N_2_ emissions and NH_3_ volatilization nor NO_3_^-^ leaching, but if included these three N flows will make reported here N balance more negative. Therefore, the partial balance presented here is underestimating N losses from the studied ecosystem and is conservative. A negative N balance for a carrot crop has been reported previously for carrots grown in organic soils [53].

It is logical to assume that fertilized carrots acquire N from the mineralized N in the soils during the growing season, at least in similar amounts to unfertilized carrots, which incorporated 176 kg N ha^−1^ into their biomass (Table 2). Based on the difference between unfertilized and fertilized treatments, we can estimate that under all but the F_400_ treatment, at least ~100% of the available soil N was incorporated into the carrots, whereas under the F_400_ treatment, only about 80% was incorporated. All additional N was probably acquired from the soils, with a ~20-fold reduction in the inorganic soil N pool, from ~ 55 kg N ha^−1^ before planting to ~2–3 kg N ha^−1^ after harvest (Table 2). Most of the 120–285 kg N ha^−1^ that was incorporated into the aboveground biomass is returned to the soil due to decomposition, as the aboveground biomass is left in the field after harvest and incorporated into the soil by postharvest tillage (Table 2).

The negative N balance and low N_2_O (and NO) emissions suggest the low potential loss of N from the fertigated carrot crops grown on mineral soils under the Mediterranean climate. On the other hand, the close to zero N balance under the F_400_ treatment indicates that an application rate of ~400 kg N ha^−1^, which is still widespread in Asia [68, 69], is close to the threshold after which a sharp increase in N losses can be expected owing to the creation of surplus N [70].

## 5. Conclusions

The partial N balance presented here, despite limitations, indicates high NUE in the studied agroecosystem. The low and uniform NO and N_2_O emissions measured in this study, regardless of increasing N fertilization, are examples of the lack of responsiveness of these emissions to fertilization. Our results indicate the need for more field-based assessments of soil NO and N_2_O emissions to ascertain the contribution of dryland agriculture to the radiative balance of the atmosphere. The lack of any significant increase in the yield or quality of the crop with fertilization at rates >100 kg N ha^−1^ indicates that the optimum fertilization rate is 100–150 kg N ha^−1^. This fertilization rate will provide safety margins for farmers, with no environmental harm, under the management practices studied here.

## Supporting information

S1 File (DOCX)
