# Supplementary material for: Soil nitric and nitrous oxide emissions across a nitrogen fertilization gradient in root crops: A case study of carrot (Daucus carota) production in Mediterranean climate
Source: PLoS One. 2023 Oct 26;18(10):e0287436. doi: 10.1371/journal.pone.0287436 (PMC10602284; doi:10.1371/journal.pone.0287436)
Supplement: S1 File — (DOCX) [file pone.0287436.s001.docx]

Supplemental information for:

Soil nitric and nitrous oxide emissions across a nitrogen fertilization gradient in root crops: A case study of carrot (*Daucus carota*) production in Mediterranean climate.

Elided Lumor J., Udi Zurgil, and Ilya Gelfand^*^

French Associates Institute for Agriculture and Biotechnology of Drylands, Jacob Blaustein Institutes for Desert Research, Ben-Gurion University of the Negev, Sede Boqer Campus, Midreshet Ben-Gurion 8499000, Israel

^*^Corresponding author.
E-mail address: igelfand@bgu.ac.il (I. Gelfand).
ORCID: 0000-0002-8576-0978.

Supplemental information contains:

Supplemental Figure S1, S2, S3, S4, and S5.

Supplemental Tables S1 and S2, S3, and S4


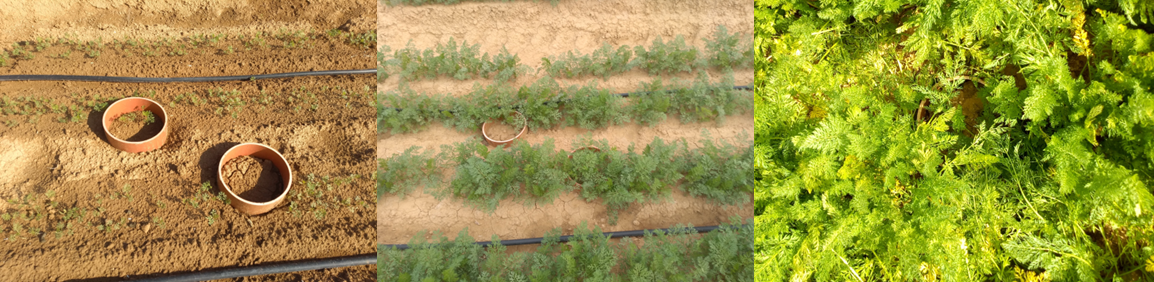


Figure S1. Chamber bases placement for soil N_2_O and NO emissions estimation. Photographs taken by authors during different stages of carrots growth. Fertigated areas visible as are wet areas on left and middle photos.

Figure S2. Seasonal patterns of (a) soil temperature (°C; 0-7 cm layer) and (b) soil water filled pore space (WFPS; % 0-25 cm layer) for different treatments during the growth season. The WFPS was calculated from GWC of soil samples and the BD (Table 1).


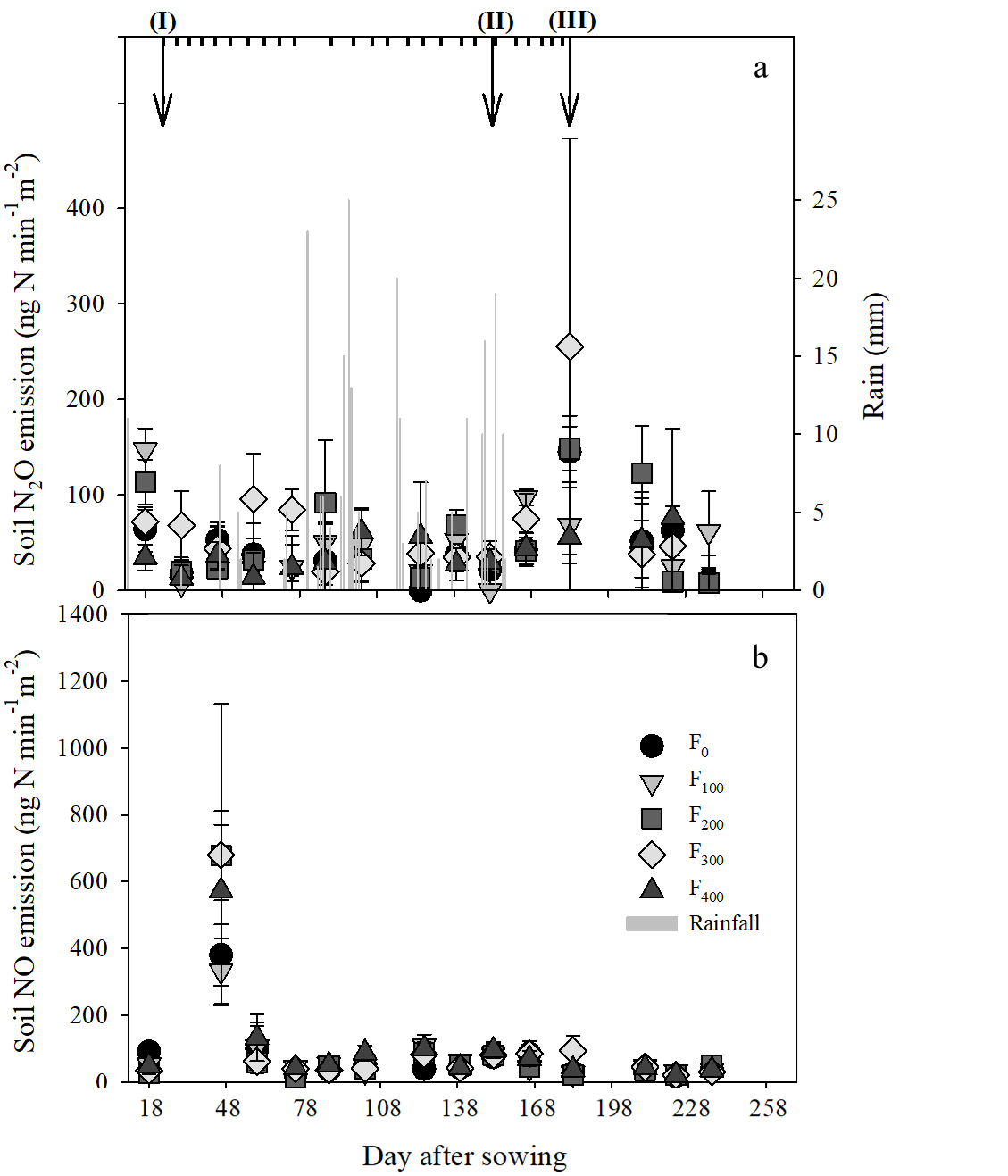


Figure S3. Daily N oxides emissions, N_2_O (**a**) and NO (**b**) from soil of the carrot field during the growth season. Start of fertilization (i), end of fertilization (ii) and harvest (iii) represented as arrows. Irrigation events are represented by marks on the upper panels. Rainfall is presented on the upper panel. Note that the y-axis scale changes between top and lower panels.


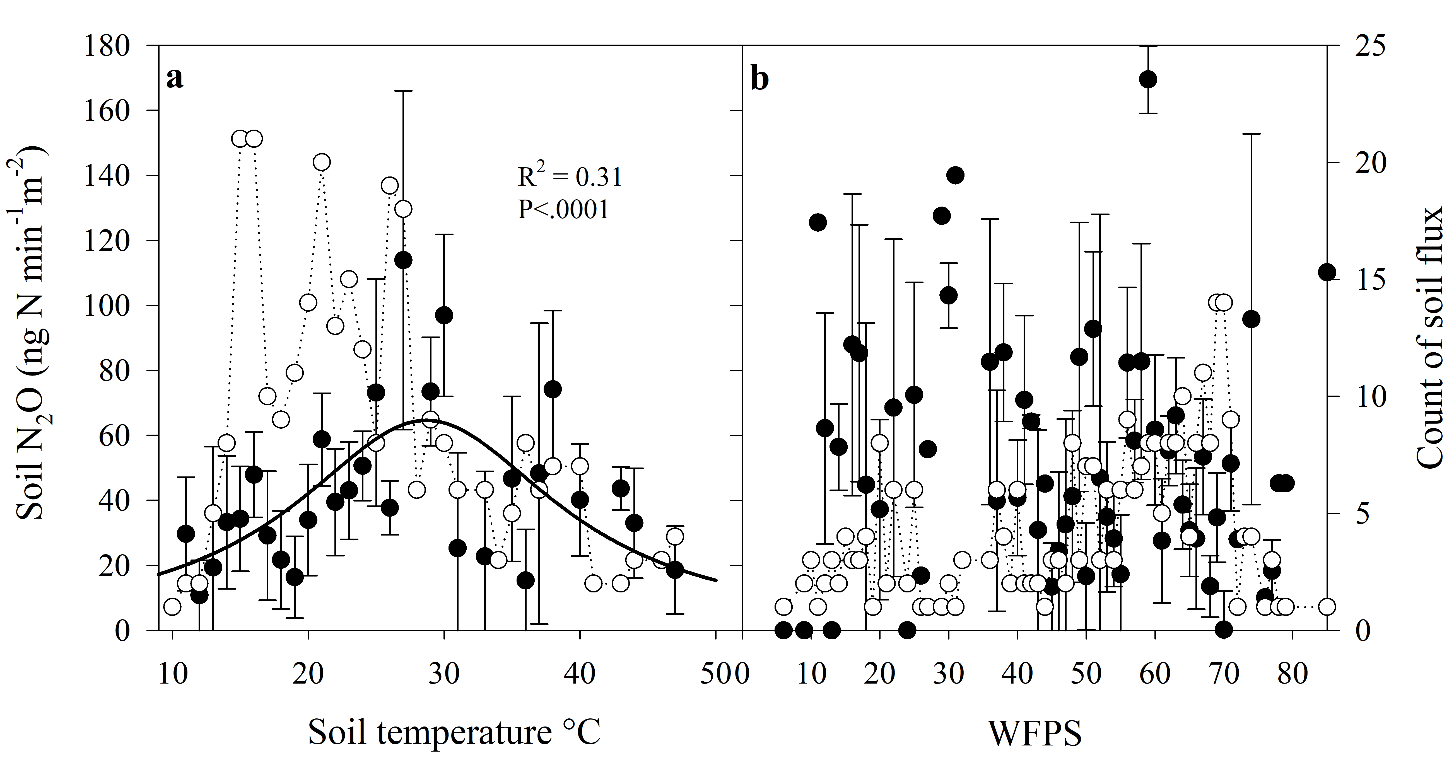


Figure S4. Relationship between temperature (a) and water-filled pore space (b) and soil N_2_O emissions aggregated to change of one degree °C or 1% WFPS; mean ± s.e. (*n* = 71) (closed symbol; soil N_2_O emission: open symbol; count of soil flux measurements).


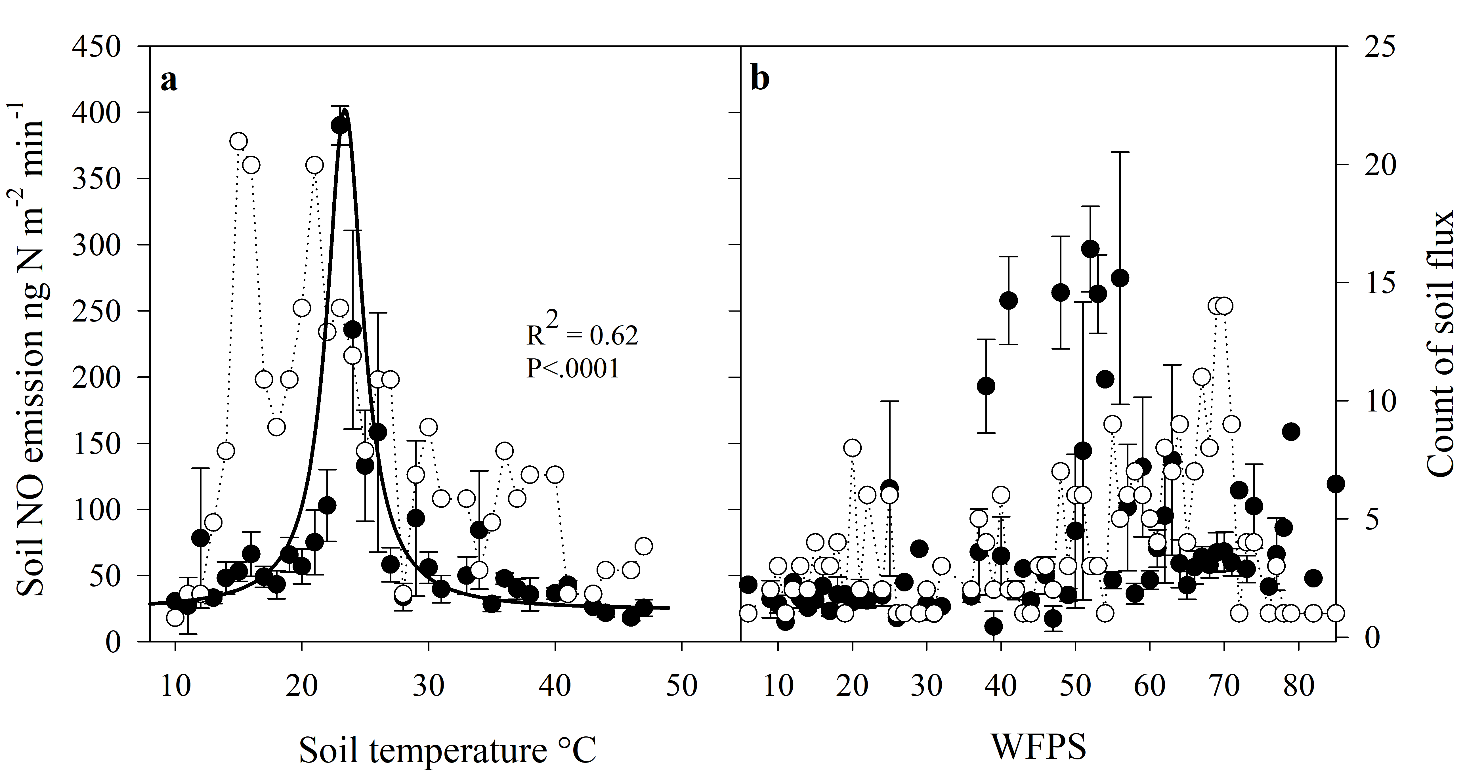


Figure S5. Relationship between temperature (a) and waterfilled pore space (b) and soil NO emissions aggregated to change of one degree °C or 1% WFPS; mean ± s.e. (*n* = 71) (closed symbol; soil NO emission: open symbol; count of soil flux measurements).

Table S1. Statistical analysis of yield size distribution for M and L carrots.

| Tukey Post-Hoc Test – **Medium** | | | | | | | | | | | | | |
| --- | --- | --- | --- | --- | --- | --- | --- | --- | --- | --- | --- | --- | --- |
|  |  |  |  |  |  |  |  |  |  |  |  |  |  |
| Treatment  *kg N ha^-1^* | |  | | 0 | | 100 | | 200 | | 300 | | 400 | |
| 0 |  | Mean difference |  | — |  | 0.280 | *** | 0.2900 | *** | 0.3283 | *** | 0.33167 | *** |
|  |  | t-value |  | — |  | 8.61 |  | 8.915 |  | 10.09 |  | 10.196 |  |
|  |  | Df |  | — |  | 25.0 |  | 25.0 |  | 25.0 |  | 25.0 |  |
|  |  | p-value |  | — |  | < .001 |  | < .001 |  | < .001 |  | < .001 |  |
| Note. * p < .05, ** p < .01, *** p < .001 | | | | | | | | | | | | | |
|  | | | | | | | | | | | | | |

| Tukey Post-Hoc Test – **Large** | | | | | | | | | | | | | |
| --- | --- | --- | --- | --- | --- | --- | --- | --- | --- | --- | --- | --- | --- |
|  |  |  |  |  |  |  |  |  |  |  |  |  |  |
| Treatment  *kg N ha^-1^* | |  | | 0 | | 100 | | 200 | | 300 | | 400 | |
| 0 |  | Mean difference |  | — |  | -0.260 | *** | -0.2833 | *** | -0.2283 | *** | -0.2117 | *** |
|  |  | t-value |  | — |  | -6.96 |  | -7.580 |  | -6.109 |  | -5.663 |  |
|  |  | df |  | — |  | 25.0 |  | 25.0 |  | 25.0 |  | 25.0 |  |
|  |  | p-value |  | — |  | < .001 |  | < .001 |  | < .001 |  | < .001 |  |
| Note. * p < .05, ** p < .01, *** p < .001 | | | | | | | | | | | | | |
|  | | | | | | | | | | | | | |

Table S2. Statistical analysis of yield size distribution for XL carrots.

| Tukey Post-Hoc Test – Extra Large | | | | | | | | | | | | | |
| --- | --- | --- | --- | --- | --- | --- | --- | --- | --- | --- | --- | --- | --- |
|  |  |  |  |  |  |  |  |  |  |  |  |  |  |
| Treatment  *kg N ha^-1^* | |  | | 0 | | 100 | | 200 | | 300 | | 400 | |
| 0 |  | Mean difference |  | — |  | -0.0650 | * | -0.0850 | ** | -0.1617 | *** | -0.1850 | *** |
|  |  | t-value |  | — |  | -3.03 |  | -3.968 |  | -7.55 |  | -8.64 |  |
|  |  | df |  | — |  | 25.0 |  | 25.0 |  | 25.0 |  | 25.0 |  |
|  |  | p-value |  | — |  | 0.040 |  | 0.004 |  | < .001 |  | < .001 |  |
| 100 |  | Mean difference |  |  |  | — |  | -0.0200 |  | -0.0967 | ** | -0.1200 | *** |
|  |  | t-value |  |  |  | — |  | -0.934 |  | -4.51 |  | -5.60 |  |
|  |  | df |  |  |  | — |  | 25.0 |  | 25.0 |  | 25.0 |  |
|  |  | p-value |  |  |  | — |  | 0.881 |  | 0.001 |  | < .001 |  |
| 200 |  | Mean difference |  |  |  |  |  | — |  | -0.0767 | * | -0.1000 | *** |
|  |  | t-value |  |  |  |  |  | — |  | -3.58 |  | -4.67 |  |
|  |  | df |  |  |  |  |  | — |  | 25.0 |  | 25.0 |  |
|  |  | p-value |  |  |  |  |  | — |  | 0.012 |  | < .001 |  |
| 300 |  | Mean difference |  |  |  |  |  |  |  | — |  | -0.0233 |  |
|  |  | t-value |  |  |  |  |  |  |  | — |  | -1.09 |  |
|  |  | df |  |  |  |  |  |  |  | — |  | 25.0 |  |
|  |  | p-value |  |  |  |  |  |  |  | — |  | 0.810 |  |
| 400 |  | Mean difference |  |  |  |  |  |  |  |  |  | — |  |
|  |  | t-value |  |  |  |  |  |  |  |  |  | — |  |
|  |  | df |  |  |  |  |  |  |  |  |  | — |  |
|  |  | p-value |  |  |  |  |  |  |  |  |  | — |  |
| Note. * p < .05, ** p < .01, *** p < .001 | | | | | | | | | | | | | |
|  | | | | | | | | | | | | | |

Table S3. Skewness and coefficient of variation of soil N_2_O and NO emissions.

| Fertilization level | NO | | N_2_O | |
| --- | --- | --- | --- | --- |
|  | skewness | C.V. | skewness | C.V. |
| *kg N ha^-1^* |  |  |  |  |
| 0 | 3.2 | 1.1 | 0.9 | 1.6 |
| 100 | 3.2 | 1.3 | 0.4 | 1.4 |
| 200 | 3.8 | 2.0 | 1.3 | 1.4 |
| 300 | 6.0 | 2.7 | 1.3 | 1.4 |
| 400 | 4.4 | 1.7 | 0.8 | 2.1 |
|  |  |  |  |  |

Table S4. Percent of positive, negative, and zero fluxes of soil N_2_O and NO emissions (total 300 individual soil fluxes of N_2_O and NO were measured).

| N oxide | Positive | Negative | Zero |
| --- | --- | --- | --- |
|  | *%* | | |
|  |  |  |  |
| N_2_O | 62 | 8 | 30 |
| NO | 99 | 0 | 1 |
|  |  |  |  |
